# Research hotspots and trends on illicit drugs: a bibliometric and visualized analysis

**DOI:** 10.3389/fmed.2025.1583173

**Published:** 2025-05-30

**Authors:** Yinyu Chen, Gaolin Zheng, Xinyan Yang, Guangmei Wu, Qianyun Nie, Peng Zhang

**Affiliations:** ^1^Key Laboratory of Tropical Translational Medicine of Ministry of Education, Hainan Medical University, Haikou, China; ^2^Department of Forensic Medicine, School of Basic Medicine and Life Sciences, Hainan Medical University, Haikou, China; ^3^Department of Pathology, School of Basic Medicine and Life Sciences, Hainan Medical University, Haikou, China

**Keywords:** illicit drugs, bibliometrics, visual analysis, web of science, CiteSpace

## Abstract

**Objective:**

To explore the current status and developmental trend of drug research in the international arena through bibliometric methods and visualization analysis.

**Methods:**

In this study, drug-related articles published from 2015 to 2024 in the core collection of Web of Science databases were analyzed, and bibliometric and visualization analyses of annual publication volume, countries (regions), institutions, journals, and keywords were achieved using CiteSpace v.6.2.R7 software.

**Results:**

A total of 5,797 publications on drug research were included between 2015 and 2024, with the annual publication volume progressively increasing on an annual basis. Among the 117 publishing countries (regions), the United States published the most articles, with 1,534 publications, followed by the United Kingdom with 611 articles. Additionally, the literature was sourced from 1,441 journals, with a total of 1,398 publications in the top 10 journals. DRUG TESTING AND ANALYSIS ranked first with 259 articles. Finally, keyword clustering and emergence analysis revealed that current research hotspots were concentrated in the areas of drug abuse, new psychoactive substances, synthetic drugs, and wastewater treatment.

**Conclusion:**

The volume of drug-related research publications is steadily increasing globally. However, there is a pressing need to further strengthen global collaboration and interdisciplinary research, as well as to promote the development of a broader international scientific research network. In particular, advanced technological approaches and policy strategies must be explored to address the global challenges posed by drug-related issues, particularly in the detection, management, and prevention of synthetic drugs. The enhancement of data sharing, technological exchange, and collaborative actions among nations plays an instrumental role in the establishment of a more efficient and coordinated global drug governance system, better equipping the international community to address the threats posed by drugs to public health and social security.

## 1 Introduction

As is well documented, drugs are defined as narcotic drugs or psychotropic substances capable of eliciting addiction, which are subject to state control. Common drugs encompass opium, heroin, morphine, methamphetamine (meth), marijuana, and cocaine, among others (1). Additionally, novel psychoactive substances (NPS) refer to recently developed substances of abuse that have garnered extensive popularity in recent years. Notably, these substances exhibit greater diversity and chemical complexity compared to traditional drugs (2).

Globally, drug use imposes significant social, personal, and economic burdens. It can lead to severe health complications, such as organ damage, mental health disorders, addiction, and overdose risks. Additionally, it can trigger the breakdown of the family and social discrimination. Moreover, it poses a substantial challenge to law enforcement and public health, given the rising risk of criminal activities such as illicit drug production, trafficking, abuse, organized crime, and violence, as well as the transmission of infectious diseases. These factors collectively jeopardize societal security and development. Furthermore, the number of drug-related deaths has surged over the past decade, highlighting the growing global problem of drug abuse.

Numerous studies investigating drug-related topics have been published on a global scale, exhibiting a consistent upward trend in terms of the number of publications. Conversely, studies that systematically analyze drug literature are scarce. Consequently, it is imperative to gain a more profound understanding of the state of drug-related research and its cutting-edge trends. Moreover, it is essential to timely identify the current status and prominent areas of focus in international drug research through the use of visualization techniques. These techniques can offer a reliable foundation for future drug research (3). Thus, CiteSpace v.6.2.R7 software was employed to conduct a bibliometric and visual analysis of drug-related articles in the Web of Science database to summarize the current status of research in the field of drug knowledge and analyze future developmental trends (4).

## 2 Data and methods

### 2.1 Data sources

The data presented herein were derived from a sample of studies based on relevant drug literature across all databases of the Core Collection of Web of Science. The following basic search pattern and search formula were employed: TS = “Illicit drugs” OR “Hard drugs” OR “Street drugs” OR “Recreational drugs” OR “Addictive drugs” OR “Psychoactive substances” OR “Designer drugs,” with a publication date range of January 1, 2015 - August 29, 2024, and a literature type selection of “Article” and “Review Article,” with language restricted to “English.” The search yielded a total of 8,096 articles. To ensure that the literature was drug-related, two forensic science graduate students individually screened the literature, with discrepancies adjudicated by a third forensic science professional (5). A total of 2,299 documents unrelated to drug research were manually excluded, resulting in the inclusion of 5,797 documents. The search results were saved as a plain text file in the format of “full record with cited references.”

### 2.2 Data analysis

In the present study, the CiteSpace v.6.2.R7 visualization software was utilized to map scientific knowledge. Relevant literature data were imported into the CiteSpace information visualization software for processing, followed by literature data cleaning, encompassing excluding duplicates and uniformly replacing articles published in Hong Kong, Macao, and Taiwan with “P. R. CHINA” (China) (6). Subsequently, information node types, such as keywords, authors, institutions, countries (regions), journals, citation frequency, etc., were analyzed and processed to obtain the visual econometric results. The research progress and trends in drug-related studies were presented through the establishment of a systematic knowledge network.

## 3 Analysis of the basic situation of the study

### 3.1 Analysis of the annual volume of publications

**Figure 1:**
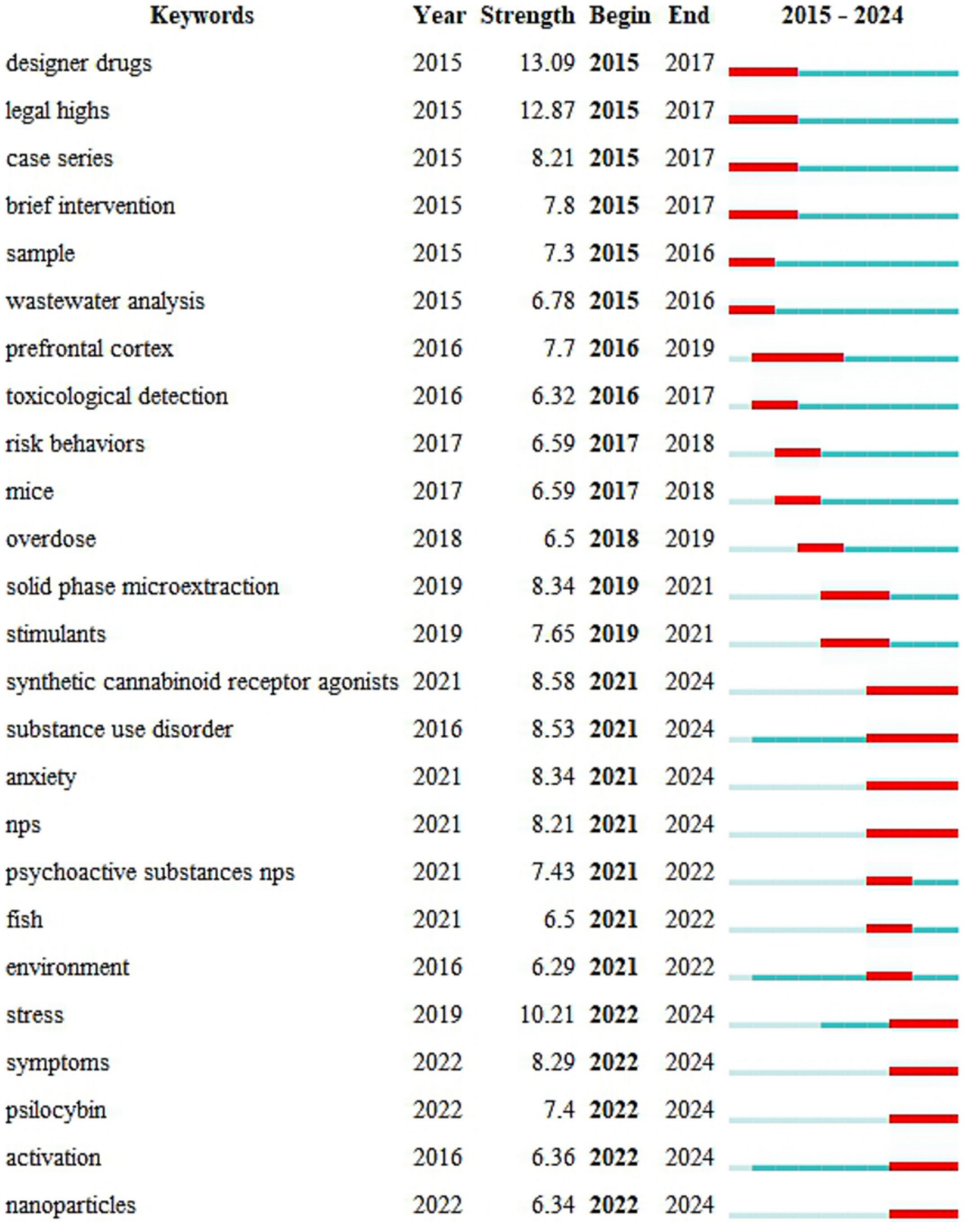
A trend of the number of illicit drugs between 2015 and 2024.

As illustrated in Figure 1, a total of 5,797 drug-related publications were identified from 2015 to 2024, exhibiting a consistent annual growth trajectory, with an average of 579.7 publications annually. Notably, the year 2021 stood out as a particularly prolific year, with 702 articles published. The relationship between the number of publications and time in the field of drug research reflects not only the degree of activity in the field but also the degree of attention received by the academic community (7). Overall, the results demonstrated that drug-related research remains an active area in the international scientific community, with expected continued growth and interest in the future.

### 3.2 Analysis of countries (regions) of issuance

A total of 117 countries (regions) published drug-related articles, among which 18 countries (regions) published over 100 articles, with an average of 49.54 articles per country (region). As outlined in Table 1, the United States (USA) ranked first with 1,534 publications, accounting for 26.46% of the total number of publications, followed by England (611 publications, 10.54%), ITALY (522 publications, 9%), PEOPLES R CHINA (China, 516 publications, 8.9%), and AUSTRALIA (516 publications, 8.9%). While Slovenia published merely 25 articles, it exhibited the highest centrality (0.97) and cooperation (11), followed by Cyprus, with 12 articles, exhibiting a centrality of 0.83 and cooperation of 9. Interestingly, the top five countries (regions), in terms of the number of published articles, demonstrated lower centrality and cooperation.

**Table 1:** Top 10 countries (regions) in terms of number of publications.

NO.	Country (region)	Number of publications (articles)	Centrality	Degree of cooperation
1	United States (USA)	1,534	0.1	4
2	England (ENGLAND)	611	0	1
3	Italy (ITALY)	522	0.03	2
4	China (PEOPLES R CHINA)	516	0.2	2
5	Australia (AUSTRALIA)	427	0	1
6	Brazil (BRAZIL)	356	0.07	3
7	Germany (GERMANY)	354	0.03	2
8	Spain (SPAIN)	347	0.1	3
9	Canada (CANADA)	340	0.03	2
10	France (FRANCE)	223	0.25	7

### 3.3 Analysis of research institutions

A co-occurrence analysis of institutions and the construction of a co-occurrence network of institutions with collaborative relationships revealed that a total of 420 institutions published drug-related research, of which seven institutions published over 100 articles. Among the top 10 institutions (Table 2), the University of London had the most publications with 190 articles, followed by the University of California System (159 articles), Institut National de la Sante et de la Recherche Medicale (124 articles), King’s College London (124 articles), University of British Columbia (124 articles), and University of London, King’s College London (124), University of British Columbia (109), Sapienza University of Rome (102), National Institutes of Health (NIH) - USA (94), University of Hertfordshire (85), and Universidade do Porto (85). The State University of New York (SUNY) System and the University of Queensland demonstrated relatively higher centrality and collaboration within the network.

**Table 2:** Top 10 institutions in terms of number of publications.

No.	Organization	Country of origin	Number of publications/articles	Centrality	Degree of cooperation
1	University of London	United Kingdom of Great Britain and Northern Ireland	190	0.1	4
2	University of California System	United States of America	159	0.19	5
3	Institut National de la Sante et de la Recherche Medicale (Inserm)	French	124	0.1	5
3	King’s College London	United Kingdom of Great Britain and Northern Ireland	124	0.03	3
5	University of British Columbia	Canadian	109	0.08	5
6	Sapienza University Rome	Italy	102	0.11	3
7	National Institutes of Health (NIH) - USA	United States of America	94	0.23	4
8	University of Hertfordshire	United Kingdom of Great Britain and Northern Ireland	85	0.09	6
8	Universidade do Porto	Portugal	85	0	1
10	University of Queensland	Australia	82	0.54	6

### 3.4 Analysis of issuing journals

A total of 1,441 journals published articles related to drug research. The top 10 journals in terms of number of publications are detailed in Table 3. Importantly, these 10 journals accounted for 1,398 publications, corresponding to 24.12% of the total publications. The journal with the highest number of articles was *DRUG TESTING AND ANALYSIS* (259 articles), followed by *FORENSIC SCIENCE INTERNATIONAL* (238 articles), *JOURNAL OF ANALYTICAL TOXICOLOGY* (1 69 articles), *SCIENCE OF THE TOTAL ENVIRONMENT* (167 articles), *DRUG AND ALCOHOL DEPENDENCE* (150 articles), and *FORENSIC TOXICOLOGY* (112 articles). Among the top 10 journals, *SCIENCE OF THE TOTAL ENVIRONMENT* had the highest impact factor of 9.8, whereas *SUBSTANCE USE & MISUSE* had the lowest (2.0). As anticipated, the top 10 journals were of high quality, with most being in the Q1 and Q2 quartiles in the JCR rankings.

**Table 3:** Top 10 journals in terms of number of publications.

No.	Periodicals	Country of origin	Number of publications/articles	IF	JCR Partitioning
1	DRUG TESTING AND ANALYSIS	United Kingdom of Great Britain and Northern Ireland	259	2.9	Q2
2	FORENSIC SCIENCE INTERNATIONAL	the Netherlands	238	2.2	Q2
3	JOURNAL OF ANALYTICAL TOXICOLOGY	United States of America	169	2.5	Q1
4	SCIENCE OF THE TOTAL ENVIRONMENT	the Netherlands	167	9.8	Q1
5	DRUG AND ALCOHOL DEPENDENCE	United States of America	150	4.2	Q1
6	FORENSIC TOXICOLOGY	Japanese	112	2.2	Q1
7	SUBSTANCE USE & MISUSE	United States of America	84	2.0	Q4
8	FRONTIERS IN PSYCHIATRY	Switzerland	76	4.7	Q2
9	JOURNAL OF PHARMACEUTICAL AND BIOMEDICAL ANALYSIS	the Netherlands	72	3.4	Q2
10	CLINICAL TOXICOLOGY	United Kingdom of Great Britain and Northern Ireland	71	3.3	Q1

### 3.5 Analysis of authors of publications

**Figure 2:**
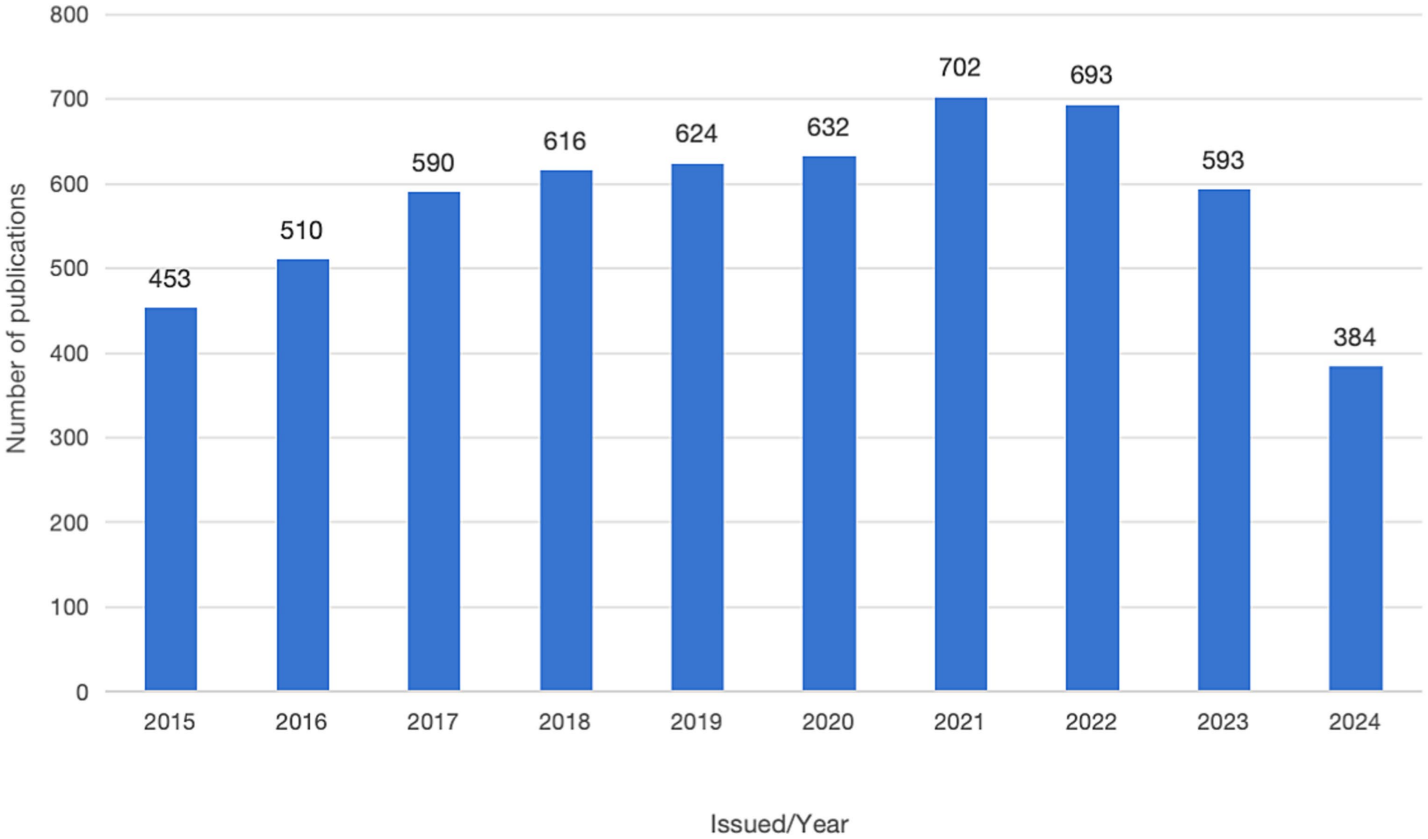
Authors collaborative networks for illicit drugs. The size of the node represents the number of articles published by the author, with larger nodes corresponding to a higher number of published articles. The color of the circle with the node represents the year of publication. The connecting line between the dots represents cooperation, while its thickness indicates the strength of the cooperation, and the color corresponds to the time when the nodes first co-occurred.

A total of 530 authors published articles during the period 2015–2024, with 14 publishing over 30 papers. Meyer MR had the highest number of publications (72). However, the research collaboration network was weak in terms of international collaboration (Figure 2). As illustrated in Figure 2, the network consists of 530 authors, 749 connections, and a network density of 0.0053. Of note, only 17 authors displayed centrality values greater than 0.1, and only seven authors had cooperation values exceeding 10. These data collectively suggest that the number of high-yield and high-impact scientific collaborators at the international level is relatively limited at present and that researchers are predominantly individuals or small-scale teams, lacking a broad international cooperation network.

### 3.6 Analysis of cited literature

The top 10 most frequently cited articles in the screened literature are summarized in Table 4, with the most frequently cited article being “New Psychoactive Substances: Challenges for Drug Surveillance, Control, and Public “Health Responses” (88 citations), published by Peacock A in Lancet. Among the top 10 co-cited documents, those related to new psychoactive substances were the most cited, followed by synthetic drugs. This finding indicates that current research hotspots are predominantly focused on these two types of drugs.

**Table 4:** Top 10 cited literature in the field of drug research.

No.	Total citations	Author	Periodicals	Total citation frequency	Centrality	Year of publication
1	New psychoactive substances: challenges for drug surveillance, control, and public health responses	Peacock A	LANCET	88	0.05	2019
2	Pharmacological characterization of designer cathinones in vitro	Simmler LD	BRIT J PHARMACOL	81	0.62	2013
3	Critical review on the stability of illicit drugs in sewers and wastewater samples	McCall AK	WATER RES	76	0.5	2016
4	The toxicology of bath salts: a review of synthetic cathinones	Prosser JM	J MED TOXICOL	71	0.12	2012
5	Spatial differences and temporal changes in illicit drug use in Europe quantified by wastewater analysis	Ort C	ADDICTION	70	0.59	2014
6	A 3-year review of new psychoactive substances in casework	Elliott S	FORENSIC SCI INT	70	0.47	2014
7	New psychoactive substances: a review and updates	Shafi A	THER ADV PSYCHOPHARM	68	0.03	2020
8	Fentanyl, fentanyl analogs and novel synthetic opioids: a comprehensive review	Armenian P	NEUROPHARMACOLOGY	68	0.01	2018
9	Novel psychoactive substances of interest for psychiatry	Schifano F	WORLD PSYCHIATRY	67	0	2015
10	Designer drugs: mechanism of action and adverse effects	Luethi D	ARCH TOXICOL	63	0.52	2020

### 3.7 Keyword analysis

#### 3.7.1 Co-occurrence analysis of high-frequency keywords

**Figure 3:**
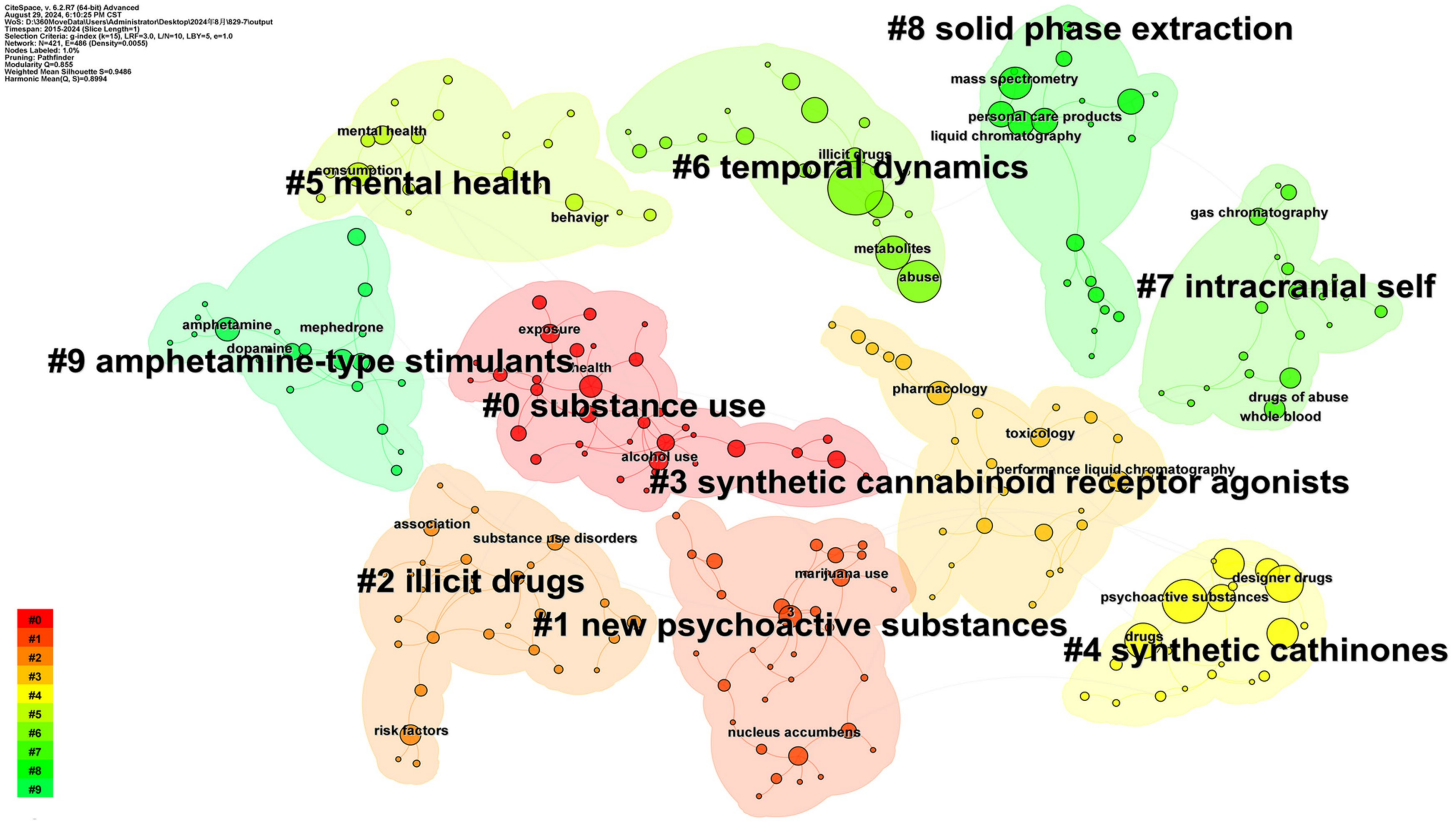
Keywords co-occurrence map of illicit drugs. The size of the node represents the frequency of keyword occurrence, with larger nodes indicating higher frequencies. The color of the circle within the node represents the year of publication. The lines between the nodes represent the links between the keywords, with thicker lines reflecting higher frequencies of co-occurrence. The color of the lines corresponds to the time of the first interaction between the nodes.

A comprehensive analysis of the keywords present within the screened drug-related articles was conducted, resulting in the construction of a knowledge graph of keyword co-occurrence (Figure 3). This knowledge graph comprises 321 nodes and 340 connecting lines. The top 10 keywords were as follows: illicit drugs (999 mentions), psychoactive substances (666 mentions), abuse (609 mentions), new psychoactive substances (591 mentions), designer drugs (459 times), identification (452 times), drugs (400 times), synthetic cannabinoids (391 times), metabolites (390 times), and substance use (364 times).

#### 3.7.2 Keyword clustering analysis

**Figure 4:**
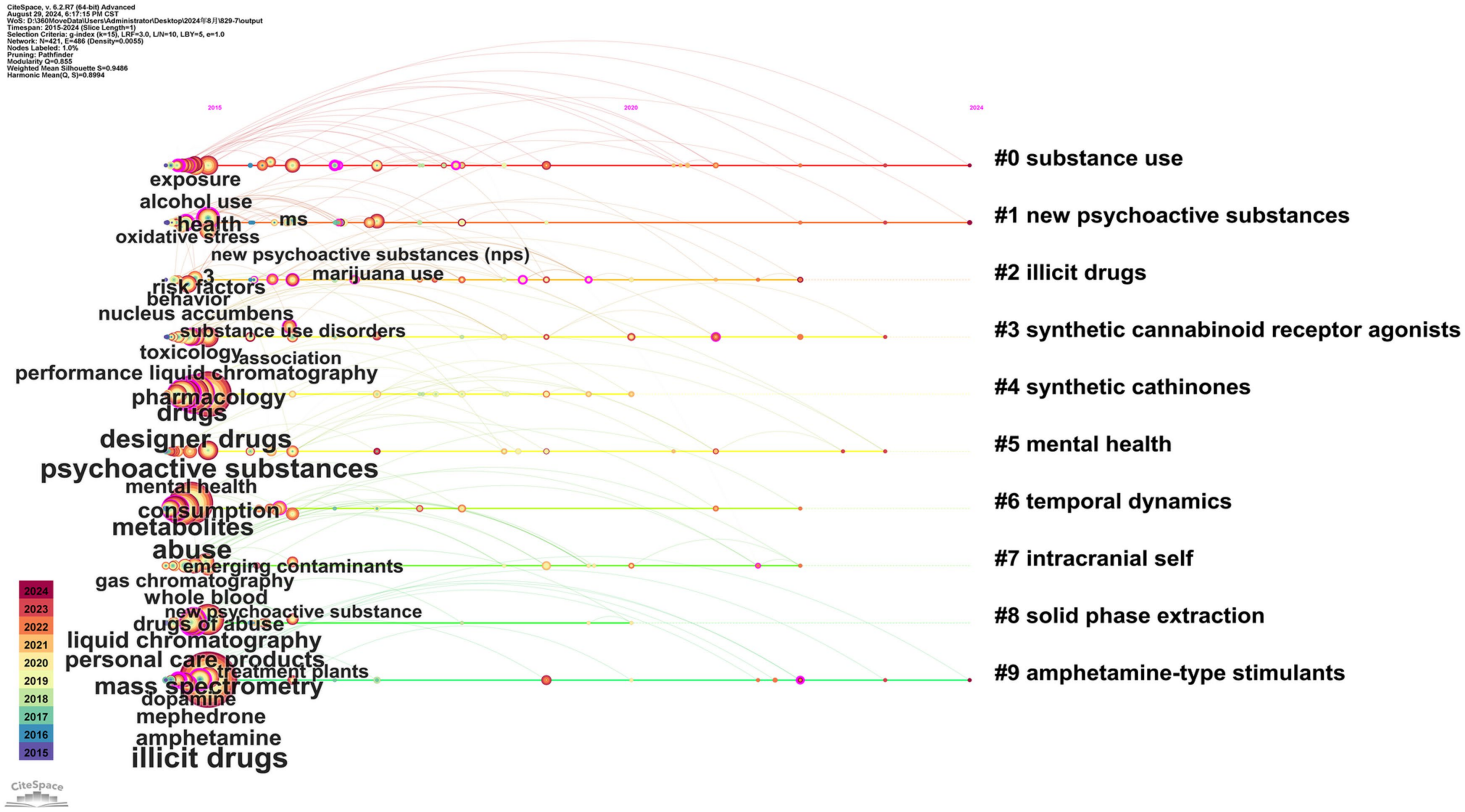
Keywords timeline view for illicit drugs. The vertical coordinate number represents the name of the cluster. The top horizontal coordinate denotes the year. The location of the node indicates the time of the first appearance of the keyword. Keywords are organized according to the time of their appearance in the cluster to which they belong, and the size of the node represents the frequency of the keyword, with larger nodes reflecting higher frequencies. The color of the circle inside the node represents the year of publication. The connecting line between the dots indicates the co-occurrence of the two keywords in one or more documents, and the color corresponds to the time of their first connection.

**Figure 5:**
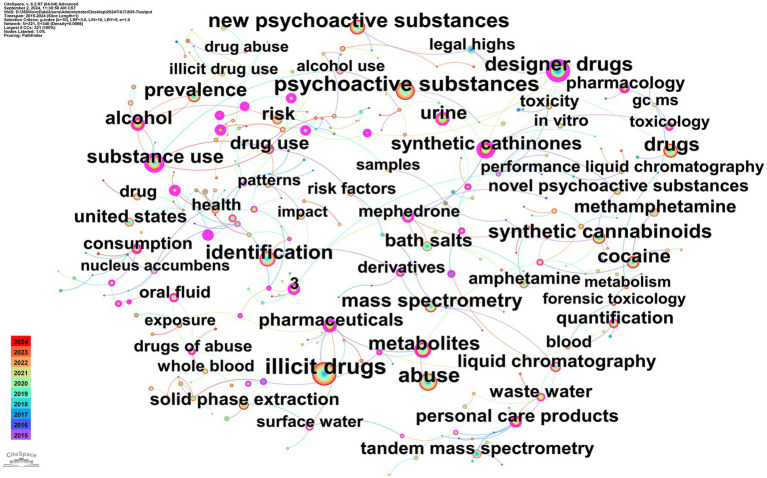
Keyword clustering map for illicit drugs.

A timeline graph of keyword interactions was constructed according to the year of publication (Figure 4) to demonstrate the dynamics of the research field. CiteSpace extracts clustering labels using the Latent Semantic Indexing (LSI) algorithm and evaluates the effectiveness of the network structure and clustering using two key metrics, namely modularity (*Q*-value) and Average Silhouette Score (*S*-value) (8). The former indicates the degree of network modularity, with a *Q*-value > 0.3 indicating effective clustering and an *S*-value > 0.7 indicating highly reliable clustering (8). The *Q*-value of 0.855 in this study indicated a well-defined structure of network clustering, while the *S*-value of 0.9486 (>0.7) indicated a satisfactory level of homogeneity within the clusters. The analysis identified 10 distinct clusters, as depicted in Figure 5. These clusters included: #0 substance use, #1 new psychoactive substances (NPS), #2 illicit drugs, #3 synthetic cannabinoid receptor agonists, #4 synthetic cathinones, #5 mental health, #6 temporal dynamics, #7 intracranial self, #8 solid phase extraction (SPE), and #9 amphetamine-type stimulants.

#### 3.7.3 Keyword emergence analysis

**Figure 6:**
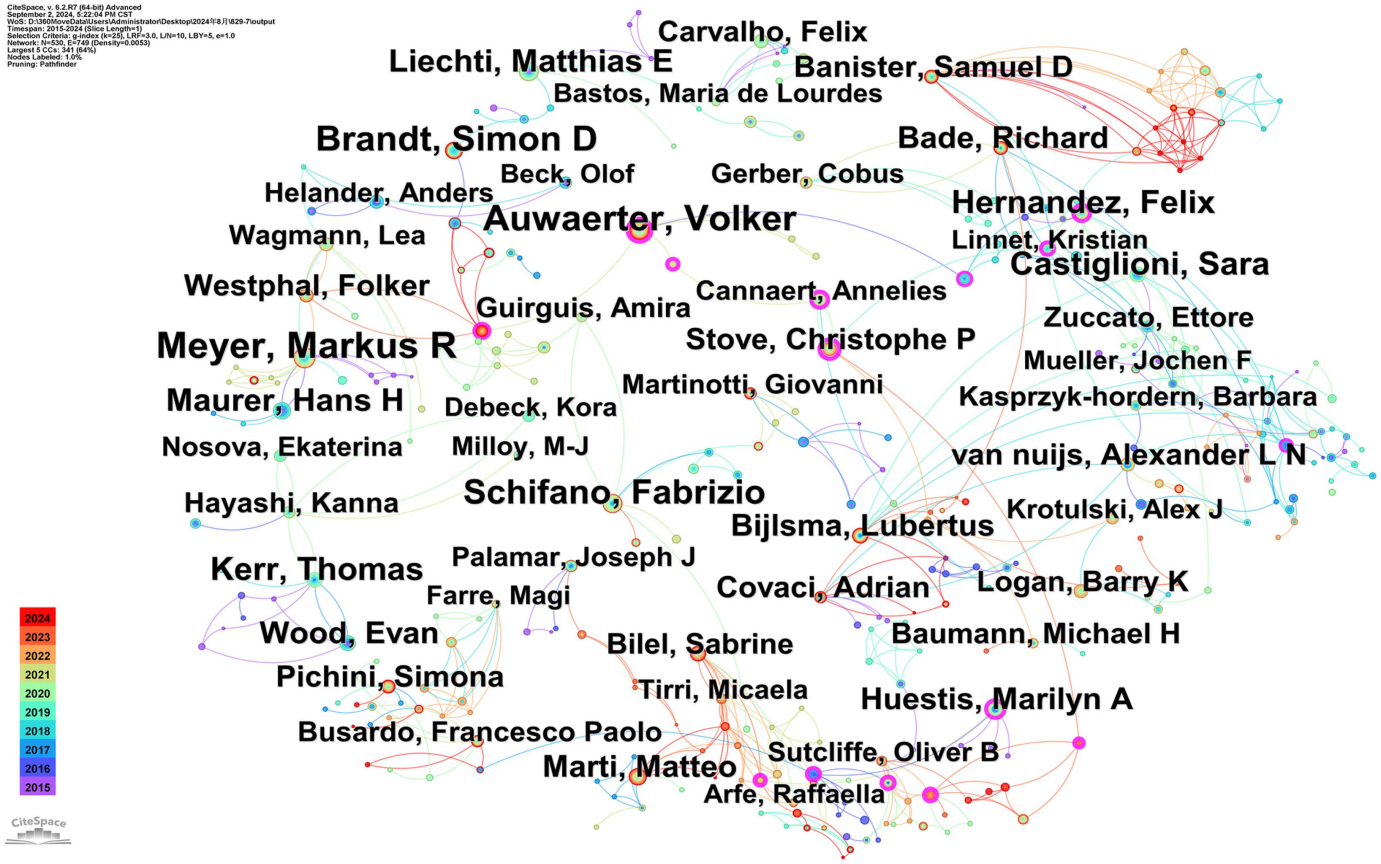
Strongest citation bursts for illicit drugs. The blue color indicates the time interval, whereas the red color indicates the duration of the citation burst.

The keyword emergence map for drug-related research (Figure 6) delineated that during the early stage (2015–2019), designer drugs, wastewater analysis, the prefrontal cortex, and toxicological detection were prominent areas of research interest. In 2021, synthetic cannabinoid receptor agonists, new psychoactive substances (NPS), and stress emerged as new research hotspots.

## 4 Discussion

The field of pharmaceutical research remains a prominent area of research in contemporary scientific discourse, playing a pivotal role in driving advancements in the field. Herein, the CiteSpace methodology was employed to conduct a scientometric analysis of the existing literature related to drug research between 2015 and 2024. This analysis involved the visualization of temporal distribution, research collaborations, research hotspots, and research trends. The results of this study uncovered a total of 5,797 documents published across 1,441 journals by 530 authors from 117 countries (regions) and 420 institutions.

### 4.1 Status of research

An analysis of the number of articles published on drug research from 2015 to 2024 revealed a general upward trend, indicative of a worldwide focus on drug-related issues and a continuous increase in research investment. As expected, the United States produced a high volume of publications. However, its centrality in the global research cooperation network remained relatively weak, signaling that although the United States has made substantial contributions to the field of drug research, it has not played a central role in global research cooperation. At the same time, the low level of cooperation between different countries indicates that research in many countries remains domestically oriented, with minimal cooperation with research institutions in other countries and regions. This lack of international collaboration may impede the global impact and innovative capacity of research conducted among individual countries. Furthermore, the mapping of institutional collaboration networks presented a fragmented pattern lacking a cohesive network. Meanwhile, the network graph uncovered a low-density network comprising 530 nodes and 749 connecting lines (density of 0.0053), suggesting relatively sparse international collaboration. This further underscores that current research remains constrained by political sensitivities and data sovereignty disputes. For instance, drug-related data from developing countries are often excluded from international research frameworks due to law enforcement confidentiality requirements. Noteworthily, only 17 authors had both domestic and international centrality values exceeding 0.1, with only seven authors having cooperation values exceeding 10. This observation further emphasizes the scarcity of high-impact scientific collaborators and cooperative teams, with the majority of research being conducted by individuals or small-scale teams. Consequently, to enhance the influence of each country in global drug research, it is imperative for researchers to strengthen exchanges and cooperation with research institutions in other countries and to promote the establishment of a more extensive international cooperation network. To break this impasse, it is imperative to establish a neutral platform akin to a “Global Drug Research Data Trust” that facilitates the standardized sharing of critical data—such as records of novel drug seizures and cross-border trafficking patterns—while ensuring robust privacy protections.

Based on the top 10 journals ranked by publication volume, research hotspots in the field of drug studies can be broadly categorized into several clusters, while also revealing a structural imbalance between technological dominance and policy lag. The *DRUG TESTING AND ANALYSIS*, *JOURNAL OF PHARMACEUTICAL AND BIOMEDICAL ANALYSIS*, and *JOURNAL OF ANALYTICAL TOXICOLOGY* journals principally publish literature on drug testing and analysis. For instance, Meyer MR, the author with the highest number of publications, employed chromatography-mass spectrometry techniques such as GC–MS, HPLC, and LC–MS for drug analysis and accurately identified the types and concentrations of drugs (9–12). In addition, researchers have developed novel detection techniques, such as optical and electrochemical sensors and nanomaterials, to address challenges posed by novel drugs. For example, Jiang X et al. fabricated graphene oxide nanocomposites based on molecularly imprinted polymers for the electrochemical sensing of new psychoactive substances (13). However, the *United Nations Office on Drugs and Crime (UNODC) 2024 World Drug Report* highlights that while detection technology research continues to dominate academic efforts, its developmental pace has been drastically outpaced by the innovative momentum of clandestine laboratories—a “technological catch-up dilemma” particularly pronounced in the synthetic cathinones domain (14).

Furthermore, researchers have focused on the metabolic pathways of drugs in the body to elucidate their pharmacological effects and toxicity (15–19). Among the most frequently cited articles, Luethi D et al. comprehensively reviewed the chemical structure, mechanism of action, and adverse effects of synthetic drugs, including stimulants, tranquilizers, dissociatives, cannabinoids, and psychedelics (20). Secondly, the field of forensic science plays a central role in drug research, as evidenced by the substantial number of publications in prominent journals such as *FORENSIC SCIENCE INTERNATIONAL* (238) and *FORENSIC TOXICOLOGY* (112). These publications conjointly highlight the importance of forensic science in combating drug-related crimes and safeguarding public health. Specifically, forensic investigations assist in determining the cause of death and assess criminal responsibility through autopsy and toxicology analyses while concomitantly focusing on drug detection and analysis to provide legal evidence in court (21–25). For example, among the top 10 most frequently cited documents, the sixth-ranked document was published in *FORENSIC SCIENCE INTERNATIONAL*, wherein the authors present collated results on the detection and/or involvement of new psychoactive substances in autopsies and criminal cases (26). However, research findings on lethal doses of NPS require an average of 3.2 years to be incorporated into the WHO risk early-warning system, underscoring systemic inefficiencies in translating academic insights into actionable policy frameworks (14).

The publication of articles in *DRUG AND ALCOHOL DEPENDENCE*, *SUBSTANCE USE & MISUSE*, *FRONTIERS IN PSYCHIATRY*, and *CLINICAL TOXICOLOGY* journals indicates a surge in research interest in the psychological and physiological aspects of drug abuse. Studies have established the effects of drugs on the brain, with a particular focus on drug use among specific populations, such as pregnant women and adolescents (27–29). Concurrently, the analysis and evaluation of drug policies in various nations have emerged as a prominent research focus, with the objective of exploring more scientific and humanized policy interventions (30–32). Numerous clinical trials have validated the effectiveness of various pharmacological and psychological interventions, while case reports have provided clinicians with invaluable practical experience and enriched the implementation pathways of intervention strategies (33–35). However, clinical intervention studies remain deficient in the multi-source data integration perspective emphasized in the Report—the majority of adolescent drug use intervention programs fail to incorporate actual composition analysis of seized drugs, resulting in systematic bias in efficacy evaluation (14).

Besides, publications in *SCIENCE OF THE TOTAL ENVIRONMENT* have drawn attention to the environmental impact of drug pollution, especially difficult-to-biodegrade drug components through wastewater discharge into natural water bodies, posing a potential threat to ecosystems and human health. The urgency of addressing this environmental issue is underscored by its inclusion in the top 10 cited literature, with numerous researchers pioneering advanced wastewater treatment technologies, such as Advanced Oxidation Processes (AOPs) and Membrane Bioreactors (MBRs), to remove drug residues from wastewater (36, 37). However, a stark disparity persists between the coverage of existing wastewater treatment technologies and the scale of drug manufacturing, with this “environmental governance deficit” escalating into an emerging ecological crisis in low-income regions (14). These contradictions underscore the imperative to construct a “technology-policy-environment” synergistic framework, such as integrating chromatographic detection standards into annexes of international drug control conventions or establishing a global fund for drug-related environmental remediation.

### 4.2 Research hot spots and frontier trends

Keywords generally reflect the core theme of articles and by comprehensively analyzing keywords in the field of drug research it is possible to summarize the research direction of the field and explore research hotspots and the current status (38, 39). Combining the keyword co-occurrence network timeline graph and emergence graph the evolutionary trajectory in drug research can be identified and research hotspots in each period can be analyzed. A comprehensive analysis of keywords in drug research revealed a predominant focus on areas such as drug abuse mental and physical health new psychoactive substances synthetic analogs and detection technologies. This analysis uncovers the multidimensional complexity of drug research exposing a fundamental contradiction rooted in the systemic disconnect between the rapidly evolving realities of substance abuse and the linearly progressing academic responses to these challenges.

The initial category is linked to drug abuse and dependence, with keywords predominantly comprising #0 substance use, #5 mental health, and #7 intracranial self-stimulation (ICSS). Consequently, research in this category is centered on the physiological, psychological, and social effects of substance abuse. Research in this field aims to elucidate the mechanisms by which drugs influence brain mechanisms and neurotransmission, with a particular focus on neuroadaptation during the development of addiction. In their seminal study, Zhang YQ et al. employed the Intracranial self-stimulation (ICSS) model to assess the abuse potential of 18 drugs. Their findings, published in Nature, demonstrated that drug-induced intracranial self-stimulation is associated with altered dopamine transporter availability in the medial prefrontal cortex and nucleus ambiguus in mice (40). Concurrently, Manu E et al. explored the effects of cannabis addiction in South African adolescents on individual behavior, cognitive functioning, and emotion regulation (41). In contrast, clinical studies have focused on treatments for substance abuse, including medication and behavioral interventions. Among these, Mindfulness-Based Interventions (MBIs) have garnered widespread attention as a treatment for a range of addictive behaviors, including alcohol consumption, tobacco use, opioid abuse, and the use of illicit substances such as cocaine and heroin (42). Garland EL et al. then provided an overview of current evaluations of MBIs in the treatment of drug addiction, focusing on their clinical outcomes and biobehavioral mechanisms. Mounting evidence suggests that microbehavioral scales can reduce substance abuse and alleviate craving by modulating cognitive, affective, and psychophysiological processes associated with self-regulation and reward processing (42). Current research trajectories demonstrate an integrated paradigm emphasizing “neural mechanisms + psychological interventions,” with growing efforts to combine neuroimaging, biomarkers, and behavioral therapies in unraveling addiction’s individual variability and critical intervention windows. Persistent controversies surround the long-term efficacy, cultural adaptability, and ethical implications of interventions, particularly regarding adolescents where the tension between punitive frameworks and therapeutic paradigms challenges policy coherence. Future investigations will likely prioritize precision intervention strategies leveraging multi-omics profiling and real-world behavioral data, alongside advancing interdisciplinary collaboration models that bridge neuroscientific insights with social determinants of addiction. Concurrently, national-level regulatory frameworks and public health policies are evolving to address addiction’s systemic pressures, though debates persist about balancing individual rights, community protection, and resource allocation in intervention architectures. Furthermore, this type of research focuses on the detrimental effects of substance abuse on public health and social order, such as increased crime rates and the depletion of social resources.

The second category comprises synthetic drugs and new psychoactive substances, which represent the focal point of contemporary drug research and the ongoing battle against drug abuse. The comprehensive keyword clustering analysis demonstrated that research in this category was predominantly centered on the following substances: #1 new psychoactive substances, #3 synthetic cannabinoid receptor agonists, #4 synthetic cathinones, and #9 amphetamine-type stimulants. The diverse and easily adaptable chemical structures of these substances engender insidious and mutable characteristics, enabling drug manufacturers to circumvent legal sanctions by altering their molecular structures. This complicates the enforcement of existing regulations and detection methods, thereby posing a significant challenge to regulation and law enforcement. Consequently, *in vivo* and *in vitro* metabolism analysis, chiral analysis, and toxicokinetic analysis have been conducted for various synthetic drugs (43–46). Current research trajectories exhibit a hallmark of deep interdisciplinary integration, where toxicology, analytical chemistry, neuroscience, and forensic science converge to propel advancements in rapid detection methodologies, metabolic pathway identification, and risk assessment frameworks for novel psychoactive substances (NPS). However, persistent ambiguities in toxicity certification criteria, inconsistencies in abuse potential classification systems, and fragmented understanding of neurodevelopmental impacts and long-term health consequences continue to trigger contentious debates between the scientific community and regulatory bodies. Future investigations are anticipated to pivot toward AI-driven predictive modeling of synthetic drug architectures and toxicological profiles, coupled with establishing real-time updatable global drug databases and interoperable international monitoring networks. Such paradigm shifts aim to achieve quantum leaps in public safety responsiveness while forging next-generation transnational governance architectures capable of countering the fluid dynamics of modern drug markets.

The third category encompasses drug detection technologies, including solid-phase extraction technology, liquid chromatography, carbon nanotubes, and other state-of-the-art detection methods. These technologies have led to substantial advancements in the sensitivity and accuracy of drug detection. Solid-phase extraction techniques have demonstrated effectiveness in the separation of drugs and other interferences, while liquid chromatography has gained widespread application in the analysis of drugs and their metabolites. A novel mixed-mode cation-exchanged magnetic inhalant, Fe3O4 @poly (ST/DVB/MA-CO OH), was prepared and used for the first time as a magnetically dispersed solid-phase extraction (MDSE) material for the highly efficient, rapid, and selective extraction of 21 drugs from wastewater by Ning H et al. (47). At the same time, carbon nanotubes have emerged as a promising detection tool owing to their high electrical conductivity and surface area. Suleman S et al. further refined these techniques, exploring their applicability to complex samples and opening up new possibilities for the standardization and simplification of drug detection (48). Recent advancements in detection technologies have prioritized practical functionality and field performance over mere sensitivity optimization, with intensified focus on user-friendly operation and rapid analysis throughput. Persistent challenges, however, surround the scalability of these innovations—particularly regarding cost-effective deployment in resource-constrained settings, standardization of evidentiary protocols for legal admissibility, and workforce training for frontline operators. Strategic priorities now emphasize bridging the technology-practice divide through AI-enhanced portable systems that balance analytical rigor with operational robustness, ultimately enabling real-time identification of novel substances while strengthening the evidentiary chain for prosecutorial applications.

The fourth category, wastewater epidemiology of drugs, refers to the monitoring of pharmaceutical compounds in wastewater and their associated environmental and societal impacts. This research domain encompasses the analysis of the temporal dynamics of drug utilization, the influence of meteorological factors on drug concentrations in wastewater, alterations in aquatic ecosystems, and the characterization of microbial diversity. By analyzing drug residues in wastewater, drug consumption can be estimated in a specific region, thereby generating real-time public health data. Notably, the first method for the simultaneous determination of 11 banned drugs and their metabolites in wastewater using ultra-performance liquid chromatography–tandem mass spectrometry (UPLC-MS/MS) with small-volume direct injection was developed by Ren H et al. (49). Furthermore, wastewater epidemiology facilitates the monitoring of environmental drug contamination and its potential impact on the ecosystem.

The keyword emergence map displayed that the emergence value of designer drugs (curated drugs) was 13.09, while that of legal highs (legal stimulants) was 12.87 in 2015–2017. These substances have been identified as research frontier topics during this period, indicating that considerable academic attention was directed toward this field. The prevalence of curated drugs and legal highs not only raises public health concerns but also poses new challenges to legal and regulatory policies. The research buzzwords of the prefrontal cortex (prefrontal cortex, PFC), overdose (addiction), etc., in 2016–2019 suggest an intricate relationship between drug abuse and brain dysfunction. Existing evidence indicates that drug addiction impairs PFC’s capacity to regulate behavior, particularly the ability to suppress impulses and make long-term decisions. In turn, this impairment hinders individuals’ ability to resist drugs and increases the risk of relapse (50). Experimental and clinical studies have unraveled the mechanisms by which drugs affect the neurochemical environment of the prefrontal cortex, leading to a decline in cognitive functioning and an increase in impulsive behaviors (51, 52). In 2021, research hotspots shifted to synthetic cannabinoid receptor agonists, novel psychoactive substances (NPS), and psychostress, among others. Of these, the emergent value of mental stress (10.21) is particularly salient, highlighting its relevance. Studies in this area mostly explored the impact of stress on the brain and body, with a particular emphasis on adolescents and children. Concurrently, research on synthetic cannabinoid receptor agonists and novel psychoactive substances has been directed toward the analysis of their chemical structures, pharmacological effects, and potential adverse effects. These studies aimed to expand our understanding of the mental and physical health of individuals in modern societies, thereby providing a scientific reference for the development of public health policies.

The findings of both keyword clustering and keyword emergence analyses indicated a high level of research interest in drug abuse, new psychoactive substances, synthetic drugs, and wastewater treatment. This suggests that drug-related problems have attracted significant attention in the field of public health and have also expanded to encompass a broader range of studies in related fields, including environmental sciences, forensic science, and social sciences. A comprehensive examination of these keywords can reveal the evolutionary trajectory of drug research, identify research hotspots, and ascertain cutting-edge trends across different time periods. This comprehensive analysis provides an invaluable reference for guiding future research endeavors.

### 4.3 Limitations of this study

While CiteSpace-based bibliometric analyses offer valuable visual insights into trends and focal points in global drug research, some limitations of this study cannot be overlooked. To begin, data sources are contingent upon specific databases and may not adequately encompass research in certain regions or fields. Secondly, variations in keyword extraction, the inherent shortcomings of software algorithms, and the influence of language and cultural differences can introduce biases into the analyses. Thirdly, the presence of homonyms and the paucity of documents in certain languages further compromise the precision and comprehensiveness of the findings.

Moreover, with the advent of novel pharmacotherapeutic agents, investigators are progressively focusing on characterizing the chemical properties and toxicological effects of new psychoactive substances (NPS), in addition to the development of more precise detection methodologies. Concurrently, artificial intelligence and machine learning techniques have been employed to predict and analyze patterns of drug abuse. Nevertheless, bibliometric methods have not yet been able to fully capture the potential of these techniques, given that research in this domain remains in its nascent stages.

At present, the study of pharmaceuticals has emerged as a prominent area of international research interest. Herein, bibliometric methods were applied to visualize and analyze the existing literature on drug-related applications from 2015 to 2024. Next, the identified research hotspots were summarized, following which future research directions were proposed. These directions include the optimization of intelligent detection technologies, the innovation of wastewater treatment technologies, and the identification and prediction of novel drug types. We further propose the adoption of suitable algorithms, such as machine learning and deep learning, to address the diverse challenges posed by these applications. This approach is expected to enhance the ability to address challenges posed by drug-related issues, improve detection efficiency and accuracy, and foster ongoing innovation within the domain of drug research.

## 5 Conclusion

In summary, this study utilized bibliometric and visualization analyses of the core collection of Web of Science databases to evaluate the current status and trends in the field of drugs on the global level. Current literature on substance abuse research predominantly focuses on addiction mechanisms, mental health impacts, and intervention strategies, with a notable rise in clinical applications of non-pharmacological approaches such as MBIs and cognitive-behavioral therapy (CBT). Meanwhile, studies targeting NPS and synthetic drugs prioritize tracking their rapidly evolving chemical architectures, metabolic pathways, and toxicity profiles, while advancing detection technologies to counter clandestine structural modifications. Emerging as a transformative tool, wastewater-based epidemiology (WBE) is increasingly deployed to map regional drug consumption patterns, synergized with ultra-sensitive analytical platforms that enhance the temporal resolution and spatial granularity of public health surveillance systems. However, a comprehensive and unified cooperative framework for drug research has not yet been established globally, and research in various countries is relatively independent. To address these challenges, it is imperative for national research institutions to play a proactive role in promoting transnational data sharing, technology exchange, and collaborative research initiatives, particularly in the domain of synthetic drug detection, management, prevention, and control. Moreover, it is crucial to explore advanced technical methodologies and the development of policy countermeasures. The critical challenge lies in aligning technological innovation with real-world exigencies—while artificial intelligence and big data analytics hold transformative potential for drug detection and usage trend forecasting, persistent tensions emerge between leveraging these tools and safeguarding ethical and privacy boundaries. Equally pressing is the imperative to dynamically recalibrate regulatory frameworks in response to the hyper-evolutionary drug landscape, where algorithmic governance models must continuously adapt to novel synthetic architectures and clandestine market dynamics without compromising civil liberties or scientific integrity. Furthermore, the establishment of a multidisciplinary, global platform for scientific research cooperation is also paramount. Such a platform should foster the formation of high-impact collaborative teams, thereby enhancing the quality and impact of scientific research outcomes. This approach will contribute to more effective responses to the global challenges posed by the drug problem, thereby offering stronger support for global public health and social security. Consequently, systematic investigations into ecological risk profiling, socioepidemiological harm surveillance, and intervention strategy optimization will emerge as a critical frontier in advancing evidence-based drug policy frameworks.

## Data Availability

The original contributions presented in the study are included in the article/supplementary material, further inquiries can be directed to the corresponding authors.
